# Contrast‐enhanced MRI synthesis using dense‐dilated residual convolutions based 3D network toward elimination of gadolinium in neuro‐oncology

**DOI:** 10.1002/acm2.14120

**Published:** 2023-08-08

**Authors:** Alexander F. I. Osman, Nissren M. Tamam

**Affiliations:** ^1^ Department of Medical Physics Al‐Neelain University Khartoum Sudan; ^2^ Department of Physics College of Science Princess Nourah bint Abdulrahman University Riyadh Saudi Arabia

**Keywords:** contrast enhanced MRI, deep learning, dilated convolution, gadolinium‐based contrast agents, glioma, medical image synthesis, neuro‐oncology, residual connection

## Abstract

Recent studies have raised broad safety and health concerns about using of gadolinium contrast agents during magnetic resonance imaging (MRI) to enhance identification of active tumors. In this paper, we developed a deep learning‐based method for three‐dimensional (3D) contrast‐enhanced T1‐weighted (T1) image synthesis from contrast‐free image(s). The MR images of 1251 patients with glioma from the RSNA‐ASNR‐MICCAI BraTS Challenge 2021 dataset were used in this study. A 3D dense‐dilated residual U‐Net (DD‐Res U‐Net) was developed for contrast‐enhanced T1 image synthesis from contrast‐free image(s). The model was trained on a randomly split training set (*n* = 800) using a customized loss function and validated on a validation set (*n* = 200) to improve its generalizability. The generated images were quantitatively assessed against the ground‐truth on a test set (*n* = 251) using the mean absolute error (MAE), mean‐squared error (MSE), peak signal‐to‐noise ratio (PSNR), structural similarity (SSIM), normalized mutual information (NMI), and Hausdorff distance (HDD) metrics. We also performed a qualitative visual similarity assessment between the synthetic and ground‐truth images. The effectiveness of the proposed model was compared with a 3D U‐Net baseline model and existing deep learning‐based methods in the literature. Our proposed DD‐Res U‐Net model achieved promising performance for contrast‐enhanced T1 synthesis in both quantitative metrics and perceptual evaluation on the test set (*n* = 251). Analysis of results on the whole brain region showed a PSNR (in dB) of 29.882 ± 5.924, a SSIM of 0.901 ± 0.071, a MAE of 0.018 ± 0.013, a MSE of 0.002 ± 0.002, a HDD of 2.329 ± 9.623, and a NMI of 1.352 ± 0.091 when using only T1 as input; and a PSNR (in dB) of 30.284 ± 4.934, a SSIM of 0.915 ± 0.063, a MAE of 0.017 ± 0.013, a MSE of 0.001 ± 0.002, a HDD of 1.323 ± 3.551, and a NMI of 1.364 ± 0.089 when combining T1 with other MRI sequences. Compared to the U‐Net baseline model, our model revealed superior performance. Our model demonstrated excellent capability in generating synthetic contrast‐enhanced T1 images from contrast‐free MR image(s) of the whole brain region when using multiple contrast‐free images as input. Without incorporating tumor mask information during network training, its performance was inferior in the tumor regions compared to the whole brain which requires further improvements to replace the gadolinium administration in neuro‐oncology.

## 1 INTRODUCTION

Magnetic resonance imaging (MRI) is a vital technique commonly used in clinics to visualize low‐contrast soft tissues and lesions in the brain. It is extensively acquired with gadolinium contrast agent administration to enhance tissues and active tumors identification.^1^ It has been reported that about one‐third of all MRI scans are acquired with the gadolinium contrast agent administration.^2^ Recently, studies have raised concerns about the safety of the use of contrast agents in medical imaging and the health impacts of gadolinium exposure.^2^, ^3^, ^4^, ^5^, ^6^ The risks of gadolinium agent administration is the accumulation of gadolinium in tissues (including the brain) and toxicity,^5^, ^6^ and development of nephrogenic systemic fibrosis.^2^, ^3^, ^4^ Thus, these studies are suggested that the gadolinium dose used should be as low as required to minimize the risks. However, reduction of gadolinium contrast doses may produce images with some key information lost.^7^ It is important to completely eliminate the use of gadolinium contrast‐based agents while preserving high‐contrast information.

MRI synthesis approaches based on deep learning currently serve as an emerging field of research in neuro‐oncology.^8^, ^9^ In particular, various deep learning‐based approaches have been investigated for contrast‐enhanced MRI synthesis toward reduction^7^, ^10^, ^11^, ^12^, ^13^ or even elimination^8^, ^14^, ^15^, ^16^, ^17^, ^18^, ^19^, ^20^, ^21^, ^22^ of gadolinium contrast agents in glioma patients. The former involves methods that propose the synthesis of full‐dose contrast‐enhanced images from their low‐dose counterparts (e.g., 10% low‐dose). On the other hand, the later involves methods that propose the synthesis of full‐dose contrast‐enhanced images from only native images.

Current research focuses on the complete elimination of gadolinium contrast agent administration via developing methods utilizing deep learning techniques to generate contrast‐enhanced MR synthetic images from contrast‐free ones. The proposed deep learning networks can be grouped into convolutional neural networks (CNNs),^13^, ^16^, ^22^ U‐Net,^16^, ^18^ Bayesian U‐Net,^14^ and generative adversarial networks.^8^, ^14^, ^15^, ^16^ ^–^ ^17^, ^19^, ^21^ Some of them use multiple MRI sequences as input,^8^, ^15^, ^16^, ^19^, ^21^, ^22^ while others utilize just a single contrast‐free MRI sequence.^17^, ^18^

Although the reported results have demonstrated the feasibility of these models for contrast‐enhanced MRI synthesis, the datasets used for training and evaluation of the models are relatively small,^8^, ^10^, ^13^, ^14^, ^15^, ^18^, ^19^, ^20^, ^21^, ^22^ containing no more than 800 patients except one study.^16^ Moreover, existing work has insufficient performance on tumor/lesion regions. In this study, we propose a 3D dense‐dilated residual U‐Net (DD‐Res U‐Net) based method for contrast‐enhanced T1‐weighted (T1) MRI synthesis on a larger scale dataset containing 1251 patients with high diversity. This larger size dataset allows us to train a deeper network and obtain improved performance. One of the advantages of dilated convolution is the effective grasping of various features at different resolution scales that permits detecting more information from small anatomical parts.

## 2 MATERIALS AND METHODS

### 2.1 Patient data

The MRI scans used in this study were collected from a public dataset, the RSNA‐ASNR‐MICCAI Brain Tumor Segmentation (BraTS) Challenge 2021 dataset.^23^, ^24^, ^25^, ^26^, ^27^ The dataset contained T1, contrast‐enhanced T1 (T1ce), T2, and T2 fluid‐attenuated inversion recovery (T2‐FLAIR) or simply “FLAIR” MRI scans of 1251 patients diagnosed with glioma. The imaging data were collected from several institutions for different scanners, magnetic fields, and imaging protocols. The images were acquired in an axial plane with various slice thicknesses (1–5 mm). Within the patient data, each MR scan was rigidly co‐registered to the T1ce and skull‐stripped. Each volumetric image had a dimension of 240 × 240 × 155 voxels and a resolution of 1 × 1 × 1 mm^3^.

### 2.2 Preprocessing

We preprocessed the data before training our model. At first, we cropped the data into smaller sizes of 180 × 180 × 128, then resized it to 128 × 128 × 128. This step allows us to remove the non‐informative voxels. For MR images, intensity normalization is critical and could adversely impact the results due to variations of signals across the MR scanners and adopting various protocols for image acquisition. Then, we applied intensity normalization by adopting the zero mean and unit variance normalization technique and scaled the data to the [0, 1] range. Normalizing the intensity before network training was found to improve the MR image synthesis results^28^ and model generalization. Finally, we randomly split the data into three sets: 64% (*n* = 800) formed the training set, 16% (*n* = 200) the validation set, and 20% (*n* = 251) the test set.

### 2.3 Network architecture

The feature maps extracted with U‐Net architecture^29^ have slightly coarse resolution due to the downsampling process in encoding the patterns. During the decoding, U‐Net uses feature maps of low‐level patterns to reconstruct target images through upsampling process. This invariance translation often compromises the results. Dilated convolutions^30^ present a potential solution to this problem and improve the resolution of results. They are capable of capturing the global context of a larger receptive field without reducing the resolution of the feature map. Similar to normal convolutional layers, dilated convolutions use the same kernel/filter size but spread out over a wider area on the image by inserting holes/gaps between its consecutive elements. The dilation rate identifies the gap size, which is recognized as an adjustable hyperparameter.

**FIGURE 1:**
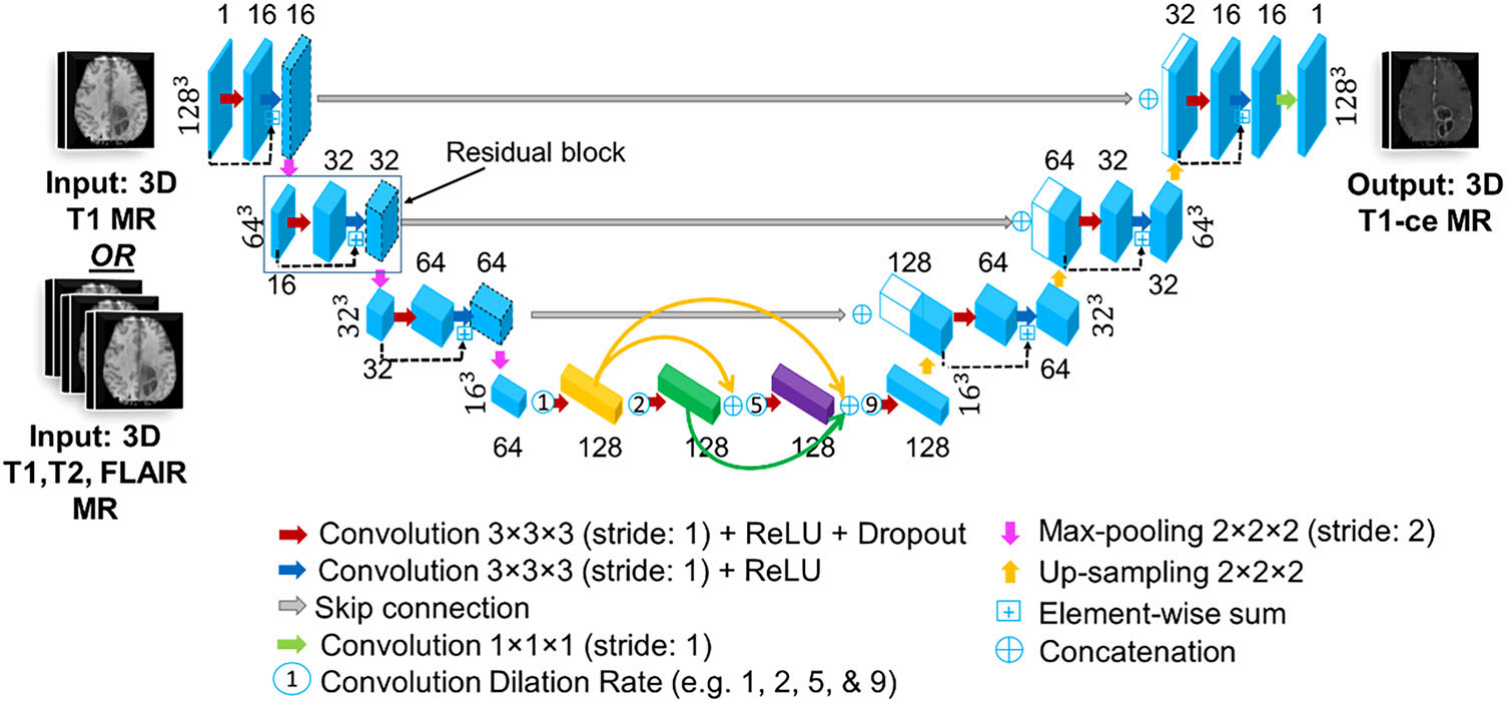
Our 3D DD‐Res U‐Net architecture for contrast‐enhanced T1 MR image synthesis from contrast‐free image(s). Each blue box represents a set of feature maps. The number on top of the box donates the extracted feature maps, and that at the left/right side of the box represents the size of the feature maps. White boxes represent copied feature maps. The arrows denote the different operations.

The network architecture of our proposed 3D DD‐Res U‐Net model is demonstrated in Figure 1. It consists of an encoder, a bottleneck, and a decoder. The encoder and decoder networks have three downsampling (using the max‐pooling operation to reduce the size of the feature maps to half) and upsampling (to increase the size of feature maps twice) multi‐scale resolution levels. The building unit of both networks is a residual block that contains two convolutional layers (3 × 3 × 3 kernel size and 1 × 1 × 1 stride), with each convolutional operation followed by a rectified linear unit (ReLU) layer^31^ to extract larger sets of low‐ to high‐level features. One of the key features is to keep the stride equal to 1 thus large overlap occurs and the model will be translational invariant (the object can be anywhere in the image). The first convolution layer in the block is always followed by a dropout layer with increasing rates at different resolution levels (0.10, 0.15, 0.20, and 0.25) to mitigate the overfitting issue. There is also an identity mapping that joins the input and output in the block. This residual skip connection^32^ is used to help train the model deeper. The encoder and decoder networks are connected via skip connections at multi‐scale resolution levels to progressively retain the original spatial resolution. The bottleneck level comprises of a densely connected sequence of dilated convolutions (dilation rates: 1, 2, 5, and 9) to capture more global context from the input without reducing the resolution of the feature map. As the dilation rate increases, a larger receptive field of the kernel can be applied while preserving resolution thus it would cover more coverage. In this densely connected level, each convolution operation is connected to all previous ones within the level. The final layer of our DD‐Res U‐Net composes of a convolutional layer (1 × 1 × 1 kernel size and 1 × 1 × 1 stride) followed by a ReLU activation function to reconstruct an output image from the feature maps that have the size of the input image.

### 2.4 Loss function

The goal is to learn a mapping function that is capable to generate contrast‐enhanced T1 images from contrast‐free MRI image(s) by minimizing the differences between the synthetic and the ground‐truth images. The loss function applied to train the proposed model in this study combines two components: pixel‐wise mean absolute error (MAE) loss $L_{MAE}$ and structural similarity (SSIM) loss *L*_SSIM_. The MAE loss is defined as $L_{\mathrm{MAE}}=\frac{1}{n}\sum_{i\;=\;1}^{n}\left|y_{i}-\;x_{i}\right|$, where *n* is the total number of voxels, $x_{i}$ and $y_{i}$ are the ground‐truth and predicted voxel values, respectively. Using pixel‐wise loss alone was found insufficient for providing satisfactory results for the task of this study. Thus, we also included SSIM loss^33^ to obtain more realistic images. The SSIM loss is defined as $L_{\mathrm{SSIM}}=\frac{1}{n}\sum_{i\;=\;1}^{n}\left|1-\mathrm{SSIM}(y_{i},\;x_{i})\right|$, where *n* is the number of slices in the volumetric MRI. The output of the $\mathrm{SSIM}(y_{i},\;x_{i})$ is a scalar between 0 and 1. The total/overall loss can be defined as the weighted sum of the two components: $L_{\mathrm{total}}=\lambda_{1}L_{\mathrm{MAE}}+\lambda_{2}L_{\mathrm{SSIM}}$, where, λ_1_ and λ_1_ are trade‐off parameters to balance the contribution of each component and their values were experimentally determined to be 1 and 5, respectively.

### 2.5 Model training

The input to the 3D DD‐Res U‐Net model is either the contract‐free T1 image or a combination of contrast‐free T1, T2, and FLAIR images with volumes of 128 × 128 × 128. At first, we trained the model multiple times to determine the optimal hyperparameters. Then it was finally trained on a training dataset (*n* = 800) using Adam optimizer,^34^ a learning rate of 0.0001, a customized loss combining MAE and SSIM loss functions, and a batch size of 1 for 50 epochs. The batch size was set to 1 due to GPU memory restriction. The model was trained to learn mapping contrast‐free image(s) to contrast‐enhanced T1 image. During the training, the model was frequently validated on the validation dataset (*n* = 200). The validation set was used to estimate the generalization error in the training and to update the hyper‐parameters. We employed an early‐stopping technique (the patience parameter set to 5) beside a dropout mechanism (with increasing rates: 10, 15, 20, and 25) as a regularization technique to minimize the likelihood of the overfitting problem. The total number of trained parameters was 2.344 million. The training was performed using Keras API (version 2.10) with a Tensorflow (version 2.10) platform as the backend in Python (version 3.10, Python Software Foundation, Wilmington, DE, USA) by using an NVIDIA Quadro M1200 4 GB GPU. After network training, it takes only a few seconds to synthesize a 128 × 128 × 128 contrast‐enhanced T1 image on new data using contrast‐free MR image(s) as input. The source code can be found at https://github.com/afiosman/dense‐dilated‐residual‐convolutions‐for‐contrast‐enhanced‐MRI‐synthesis.

### 2.6 Evaluation

The synthetic images were qualitatively and quantitatively compared to the ground‐truth on the test set (*n* = 250). Various evaluation metrics were used for comparing the images including the SSIM, MAE, mean‐squared error (MSE), peak signal‐to‐noise ratio (PSNR), normalized mutual information (NMI), and Hausdorff distance (HDD). We also trained a 3D version of the standard U‐Net^29^ to compare the powerful of our proposed model with a baseline model.

## 3 RESULTS

**FIGURE 2:**
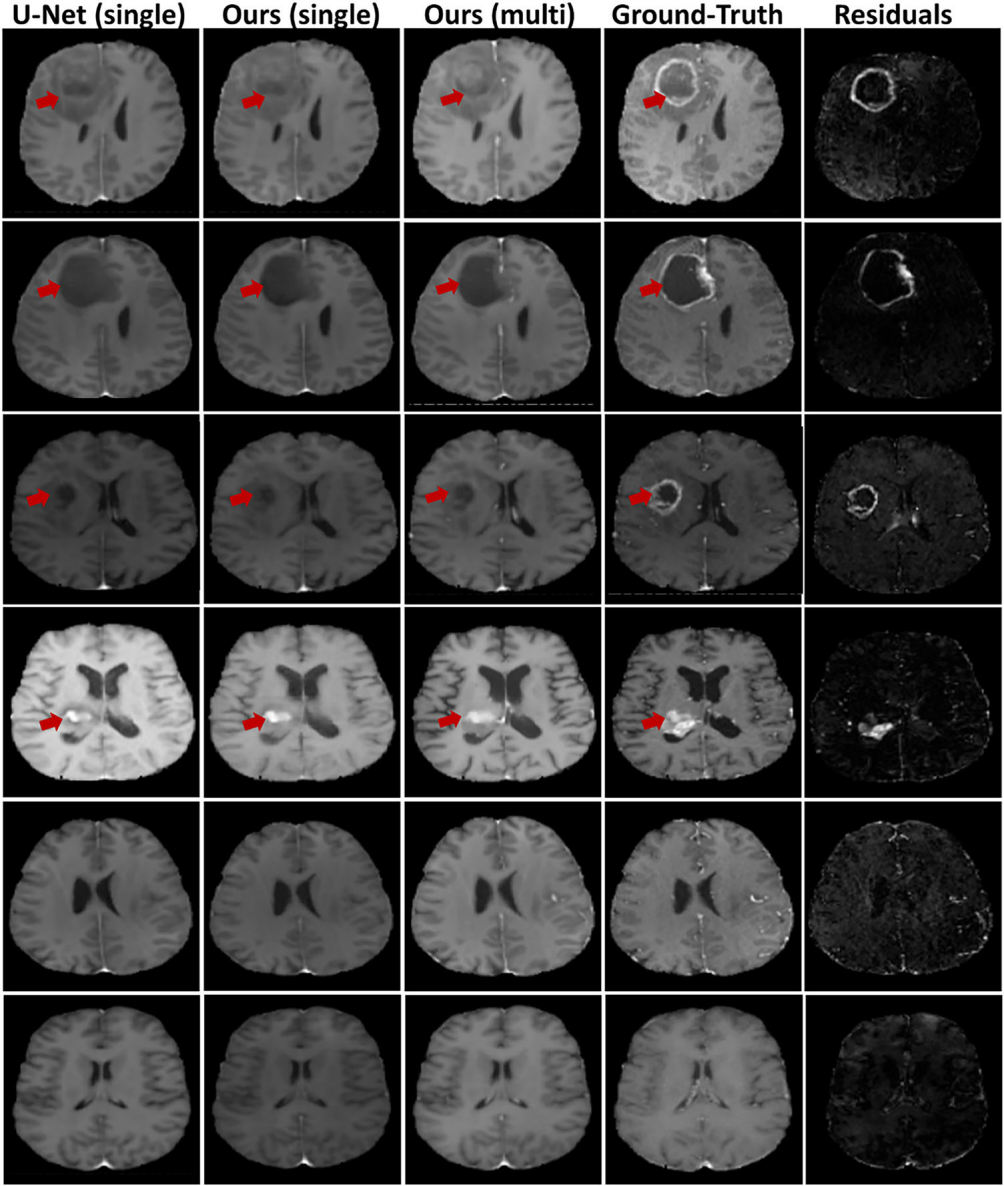
Visual assessment of our proposed DD‐Res U‐Net model for contrast‐enhanced T1 MRI synthesis and a 3D U‐Net baseline model for 6 patients on the test set. From left to right: contrast‐enhanced T1 synthetic image using only T1 as input (DD‐Res U‐Net); contrast‐enhanced T1 synthetic image using only T1 as input (DD‐Res U‐Net); contrast‐enhanced T1 synthetic image using T1, T2, and FLAIR as input (DD‐Res U‐Net); ground‐truth image; and residuals (absolute difference between synthetic and ground‐truth). Rows show results for different patients.

The results of representative test samples are presented in Figure 2. The first four rows show a subject with tumors and the last two rows display normal subjects without any tumors. Visual or qualitative assessment of the images reveals several interesting observations. First, the generated contrast‐enhanced T1 synthetic images by our model using multi‐input images (T1, T2, and FLAIR) were almost indistinguishable from their corresponding ground‐truth ones for normal brain tissues. The fine anatomical structures are preserved in the synthesized images and are nearly identical to the ground‐truth images. Voxel‐wise error profiles or residuals of the synthetic contrast‐enhanced MR images exhibited minimal intensity differences. Second, the results show that our model is more likely to over‐fit normal regions. This is due to the fact that tumor regions are limited relative to the whole brain volume. Thus, the generated images at tumor regions are inferior to that of normal regions. Third, combining multiple images as input (T1, T2, and FLAIR) seemed to improve the quality of the synthesized contrast‐enhanced T1 images. Finally, comparing our model performance to a 3D U‐Net baseline model shows that the results obtained by our model were superior to the standard U‐Net model. The U‐Net baseline model tends to under‐estimate tumor regions and critical tiny structures of the brain tissues.

The quantitative performance of the proposed model is shown in Table 1 on all patients in the test set (*n* = 251). The metrics are computed based on the brain regions. Our model consistently achieved better performance over the U‐Net baseline model in almost all metrics (PSNR, MSE, NMI, and HDD). Besides, our proposed model achieved the best when combining T1, T2, and FLAIR as input in terms of PSNR, SSIM, MAE, MSE, NMI, and HDD, which is consistent with our visual assessment.

**TABLE 1:** Quantitative comparison between our proposed DD‐Res U‐Net model and the U‐Net baseline model over the whole brain region using various evaluation metrics on the test set (*n* = 251).

Input MR	Model	Evaluation metric					
		PSNR ↑ (dB)	SSIM ↑	MAE ↓	MSE ↓	HDD ↓	NMI ↑
T1	U‐Net	29.667 ± 5.030	0.901 ± 0.070	0.018 ± 0.013	0.002 ± 0.002	2.435 ± 10.146	1.343 ± 0.086
	DD‐Res U‐Net	29.882 ± 5.924	0.901 ± 0.071	0.018 ± 0.013	0.002 ± 0.002	2.329 ± 9.623	1.352 ± 0.091
T1, T2, FLAIR	DD‐Res U‐Net	**30.284 ± 4.934**	**0.915 ± 0.063**	**0.017 ± 0.013**	**0.001 ± 0.002**	**1.323 ± 3.551**	**1.364 ± 0.089**

*Notes*: The vertical arrow's direction indicates the trend for better results and higher image qualities. Results reported as the mean ± 1 standard deviation. Bold indicates the best results.

Abbreviations: HDD, Hausdorff distance; MAE, mean absolute error; MSE, mean‐squared error; NMI, normalized mutual information; PSNR, peak signal‐to‐noise ratio; SSIM, structural similarity.

**FIGURE 3:**
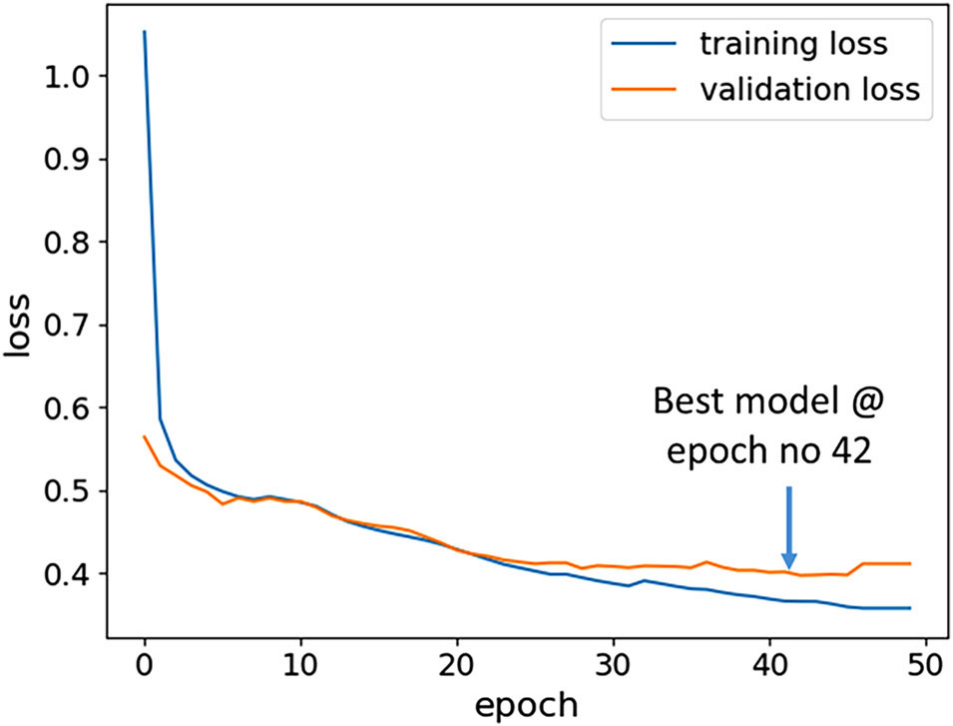
The model performance curve.

Visual assessment of the training and validation losses over the number of epochs is an effective approach to identify whether the proposed model is well‐trained or not. Figure 3 displays the learning curves of the proposed model in this study for contrast‐enhanced T1 MR image synthesis. We can observe that gap between the training and validation loss curves is minimal at earlier epochs before exhibiting an overfitting problem later. The optimal model at epoch number 42 was saved yielding the best compromise between the loss error and the gap between the training and validation curves. This also indicates the good generalizability of the model to perform well on new data.

## 4 DISCUSSION

Contrast‐enhanced T1 sequence is crucial for accurate diagnosis and treatment of glioma patients. However, recent studies have raised broad safety and health concerns about using gadolinium contrast agents in medical imaging. Here, we proposed a deep learning‐based 3D network using dilated convolutions and residual connections for contrast‐enhanced T1 image synthesis from contras‐free image(s) to eliminate the need for gadolinium contrast agent administration in neuro‐oncology. We utilized dilated convolution due to its effectiveness in grasping various features at different scales. Thus, it would permit more information from tiny anatomical structures.

We believe that proper data pre‐processing and hyperparameters optimization are more important than architectural modifications. As a result, we cropped out and resized the data to a smaller size for easier handling. In addition, we normalized and scaled the data to overcome the problem of the heterogeneous intensity of MR images due to variations among the scanners and institutional protocols. This step is essential for training the model for accurate predictions and greatly alters its performance. We experimentally identified the optimal hyperparameters for training the model for contrast‐enhanced image synthesis. We found that applying global loss functions such as MAE or MSE is ineffective for accurate contrast‐enhanced image synthesis, particularly in the tumor regions. This is because the tumor volumes are limited relative to the whole brain volume. We, therefore, adopted a customized loss function defined as a weighted sum of MAE loss and SSIM loss to improve the model performance in the tumor regions. This loss function was found to provide much better results than any individual one.

The experimental results show the feasibility of our model to generate synthetic contrast‐enhanced T1 images that are not differentiable from ground‐truth (Figure 2). The synthetic images generated by our model using multi‐input images (T1, T2, and FLAIR) were almost indistinguishable from their corresponding ground‐truth ones for normal brain tissues. The critical anatomical structures are preserved in the synthesized images. For the lesion regions, the quality of the generated images was slightly inferior to that of normal regions. The model tends to most likely underestimate the tumor region and over‐fit normal regions since tumor volumes are limited relative to the whole brain volume. It is worth highlighting that we did not incorporate tumor contour information during the network training which could significantly improve the reported results at the lesion regions. The quantitative results (Table 1) indicate that our model outperformed the 3D U‐Net baseline model trained and evaluated on the same dataset. We utilized U‐Net architecture as a baseline model because it has been successfully applied to many other tasks. The results also indicate that combining multiple images as input (T1, T2, and FLAIR) significantly improves the quality of the synthesized images.

We also compared our results to the existing state‐of‐the‐art methods in the literature. The quantitative results exhibited that our model achieved better results in terms of PSNR (30.284 ± 4.934 vs. 28.24 ± 1.26) and MAE (0.017 ± 0.013 vs. 0.029 ± 0.005) than that reported by Chen et al.^22^ in the whole brain region. The author of this work^22^ used T1, T2, and apparent diffusion coefficient map as input images to a high‐resolution fully‐convolutional network and the contrast‐enhanced T1 as the target image. Our results were also better by a large margin than that reported by Preetha et al.^16^ in terms of SSIM (0.901 vs. 0.818) for synthesizing contrast‐enhanced T1 images from T1, T2, and FLAIR using deep CNN. However, our results were inferior to that reported by Dai et al.^8^ using a unified GAN (PSNR: 29.882 ± 5.924 vs. 32.353 ± 2.525; SSIM: 0.901 ± 0.071 vs. 0.974 ± 0.059) and Xie et al.^18^ utilizing 3D Retina U‐Net and Synthesis module (SSIM: 0.901 ± 0.071 vs. 0.991 ± 0.007) in synthesizing contrast‐enhanced T1 images from contrast‐free T1 images. Both methods were trained using the same dataset pool (BRATS’2015 and BRATS’2020). Note that in Xie et al.,^18^ the author utilized contour information during their model training which greatly improves the tumor regions synthesis as well as the reported results.

There are a few limitations associated with this study. First, as the tumor regions account for a very small proportion of the whole brain, the performance of the model on these regions remains sub‐optimal. Incorporating contour information of the tumor during the network training may likely improve the performance further at tumor regions. Second, our model requires prior registration of the images for proper model training. Thus, imperfect co‐registration of the images may lead to the loss of tiny structures of the brain in the synthesized images. Finally, this study did not provide a comparison of the tumor contour volumes delineated on the ground‐truth and synthetic MR images to show whether our method can give the closest volume size to ground‐truth contour. To do so, it requires consistent manual/auto contouring of the tumor region to maintain high accuracy that was not feasible during this study.

## 5 CONCLUSION

We proposed a 3D U‐Net with dilated convolutions and residual connections to generate contrast‐enhanced T1 synthetic images from contrast‐free MR image(s) to eliminate the risks of using gadolinium contrast agents in neuro‐oncology. We utilized the largest well‐designed publicly available dataset of the BRATS’2021 challenge for this purpose. Besides, we also adopted a customized loss function combining the MAE loss and SSIM loss to improve the model performance. The experimental results showed excellent performance of our model in generating high‐quality contrast‐enhanced T1 synthetic images for normal subjects or whole brain MRI. In the tumor regions, however, the performance is far from perfect and inferior to the whole brain, our model sometimes misses or underestimates some tumors, especially lesions that are not distinct enough in the contrast‐free images. Utilizing a contrast‐free T1 image in combination with other MRI sequences as input has been shown to significantly improve the synthetic images compared to using only the contrast‐free T1 image. Comfpared to existing state‐of‐the‐art models, our method outperformed similar works in the literature and was inferior to a few ones where tumor contour information was incorporated during the network training. With further improvements of the model performance in the tumor regions, the proposed model holds great potential for clinical utility in substituting the gadolinium contrast agent administration in neuro‐oncology. Future work will concentrate on imperfect performance in tumor regions.

## AUTHOR CONTRIBUTIONS

A. O. contributed to the conception and design of the study, developing the models, drafting the revising the manuscript for important intellectual contents. N.T. contributed to writing and revising the manuscript. All authors contributed to manuscript revision and approved the submitted version.

## CONFLICT OF INTEREST STATEMENT

The authors declare no conflicts of interest.

## Data Availability

The datasets generated during and/or analyzed during the current study are available at the RSNA‐ASNR‐MICCAI Brain Tumor Segmentation (BraTS) Challenge 2021 repository, (http://braintumorsegmentation.org/). The source code is also available at https://github.com/afiosman/dense‐dilated‐residual‐convolutions‐for‐contrast‐enhanced‐MRI‐synthesis.
